# Supplementary material for: Factors that contribute to loss to follow-up in the medium term after initiation of anti-vascular endothelial growth factor therapy for neovascular age-related macular degeneration in Japanese patients
Source: PLoS One. 2025 Jun 11;20(6):e0325963. doi: 10.1371/journal.pone.0325963 (PMC12157206; doi:10.1371/journal.pone.0325963)
Supplement: Attachment — Submitted filename: review.docx [file pone.0325963.s001.docx]

This study by Sugisawa et al. investigates the factors contributing to loss to follow-up among patients undergoing anti-VEGF therapy for neovascular age-related macular degeneration (nAMD) in 16 Japanese retinal facilities. The manuscript addresses a relevant clinical issue, but several aspects require clarification and improvement to enhance its impact and scientific validity.

1. The term "loss to follow-up" is insufficiently defined. Does it strictly refer to patients who do not return for care, or does it also include those transitioning to other care facilities? Transitioning to local clinics or other centers may not constitute a true loss, as patients could still be receiving treatment. Clear criteria are essential.
2. While the retrospective design is understandable, potential biases in treatment strategies across the 16 centers must be addressed. Treatment approaches (e.g., "as needed" vs. "treat and extend") and medication choices (e.g., bevacizumab, aflibercept, ranibizumab) may vary significantly among centers. Were there notable inter-center differences in management strategies?
3. While the authors focus on ophthalmologic variables such as visual acuity and macular thickness, it is crucial to recognize that factors influencing "loss to follow-up"—such as socioeconomic status, travel distance, drug cost, insurance coverage, and caregiver presence—are likely more significant determinants in this context. Can the authors add those variables directly or indirectly into their analysis?
4. The manuscript acknowledges the COVID-19 pandemic as a factor but lacks a thorough analysis. Subgroup analysis comparing pre-pandemic and pandemic-era cohorts would offer valuable insights into its influence on follow-up rates.
5. The discussion attributes loss to follow-up to factors like patient dissatisfaction or disease severity without direct supporting evidence. Incorporating patient-reported outcomes or survey data could substantiate these claims. Is there existing evidence to support the authors' interpretations?
6. While the authors attempt to draw clinical implications, they fall short of providing actionable recommendations. For instance, how can physicians address loss to follow-up in older patients or those with worse baseline visual acuity or greater central retinal thickness? Practical strategies or interventions would enhance the manuscript's utility.
7. Ensure uniform use of terms, such as "visual acuity" and "BCVA," throughout the manuscript to maintain clarity and readability.
8. Figures 3, 5, and 7 require improvement in axis labels and font sizes for better readability. Standardizing these elements will enhance the overall presentation quality.
